# Risk Prediction of Central Nervous System Infection Secondary to Intraventricular Drainage in Patients with Intracerebral Hemorrhage: Development and Evaluation of a New Predictive Model Nomogram

**DOI:** 10.1007/s43441-022-00403-2

**Published:** 2022-04-24

**Authors:** Yanfeng Zhang, Qingkao Zeng, Yuquan Fang, Wei Wang, Yunjin Chen

**Affiliations:** Department of Neurosurgery Intensive Care Unit, Meizhou People’s Hospital, Meizhou, 514031 Guangdong China

**Keywords:** Intracerebral hemorrhage, Ventriculostomy tube drainage, CNS infection, Nomogram

## Abstract

**Background:**

Currently no reliable tools are available for predicting the risk of central nervous system (CNS) infections in patients with intracerebral hemorrhage after undergoing ventriculostomy drainage. The current study sought to develop and validate a nomogram to identify high-risk factors of CNS infection after ventriculomegaly drain placement for intracerebral hemorrhage.

**Methods:**

A total of 185 patients with intracerebral hemorrhage who underwent ventriculoperitoneal drainage were enrolled to the current study. Patients were divided into a CNS infection group (20 patients) and a non-CNS infection group (165 patients). The baseline data from both groups was used to develop and evaluate a model for predicting the likelihood of developing CNS infection after ventriculoperitoneal drain placement for intracerebral hemorrhage.

**Results:**

The finding showed that operative time, intraventricular drainage duration, postoperative temperature, white blood cell count in cerebrospinal fluid (CSF), neutrophils ratio in CSF, Red blood cell count in CSF, and glucose content in CSF were correlated with CNS infection. A nomogram for predicting the risk of CNS infection was constructed based on these variables. The c-index and the AUC of the ROC curve was 0.961, showing good discrimination. Clinical decision curve analysis indicated that the nomogram clinical application ranged between 1 and 100%. The clinical impact curve was generated to set with a threshold probability of 0.5.

**Conclusion:**

The nomogram reported in the current study can be used by clinicians to identify patients likely to have secondary CNS infections, so that clinicians can better treat these patients at earlier stages.

## Introduction

Neurosurgery central nervous system infections (NCNSIs) refer to infections either secondary to neurosurgical illness or intracranial and intraspinal disorders that require management by neurosurgery [1]. CNS infections can be divided into primary and secondary infections. The causative organisms of these infections are diverse. The main types of secondary CNS infections include open head injury, CSF leakage, and bacterial infections due to extraventricular drainage. Clinical manifestations of secondary CNS infections are often nonspecific, and the infections may lead to hyperthermia, coma and even death of patients if not diagnosed on time and if targeted treatment is not used.

Surgery of external ventricular drainage is a common treatment for intracerebral hemorrhage. However, the patient is at risk of developing a CNS infection because the drainage tube passes through the patient in and out of the skull. CNS infections can be severe, thus affecting the daily life of the patient, or can be fatal [2]. Early diagnosis for patients with intracerebral hemorrhage is limited by several factors and thus it is challenging to determine whether they have secondary CNS infection after ventriculostomy drainage. For example, early meningitis has no evident specific clinical manifestations, and CSF bacterial culture, which is the gold standard for diagnosis of CNS infection, has several disadvantages, such as low sensitivity and time-consuming culture [3]. Therefore, there is need to explore the risk factors of CNS infection after ventriculoperitoneal catheter drainage for intracerebral hemorrhage. Identification of the risk factors ensures early identification and treatment of patients with secondary CNS infection, thus improving early diagnosis rate.

A nomogram model is a tool for integrating disease-related risk factors and for determining numerical probabilities of target events with individualized ability to predict the risk of developing disease through an intuitive model display [4]. Previous studies report that nomograms are useful in predicting lymph node metastasis of colorectal cancer and survival of lung cancer [5, 6].

In the current study, risk factors for secondary CNS infections such as operative time, postoperative temperature, intraventricular drainage duration, CSF white blood cell count, CSF neutrophils ratio, CSF red blood cell count, and CSF sugar content were identified through regression analysis. A nomogram model was established based on the above risk factors for personalized prediction of the likelihood of secondary CNS infection in patients with intracerebral hemorrhage undergoing ventriculostomy.

## Materials and Methods

### Data Collection

The current study retrospectively included patients who underwent ventriculostomy due to intracerebral hemorrhage from January 2016 to August 2020 at the Meizhou People’s Hospital. Basic clinical information of the patients, including gender, age, length of surgery, duration of drain retention, postoperative temperature, and whether CNS infection occurred secondarily, were obtained from medical records. Laboratory test results of patients in the CSF were obtained, including white blood cell count, neutrophil ratio, number of red blood cells, glucose content, protein content, and chloride content in the CSF. Patients gave informed consent prior to the study. The study was approved by the ethics committee of Meizhou People’s Hospital.

Diagnosis of neurosurgery CNS infection is divided into clinical diagnosis and etiologically confirmed diagnosis. Compliance with 1–4 of the following criteria indicates a clinical diagnosis [7], compliance with 1–5 of the criteria indicates etiological diagnosis: (1) Clinical manifestations including systemic inflammatory reactions, changes in consciousness and mental status, Symptoms and signs of increased intracranial pressure, Meningeal irritation signs, and Concomitant signs or symptoms [8]. (2) Blood related tests including blood routine white blood cells > 10.0 × 10^9^/L and neutrophil ratio > 0.8. (3) Intracranial pressure and cerebrospinal fluid related examinations including intracranial pressure [7], CSF characteristics, CSF WBC count and ratio, CSF biochemistry [8], and CSF glucose content/serum glucose content [9]. (4) Imaging findings. (5) Smears and cultures of cerebrospinal fluid, incisional secretions, drains, implants, and surgical specimens: smear of specimen, head of drain, implant, and cerebrospinal fluid positive microbiological culture is the gold standard for diagnosis, except in cases of contamination and colonization. Mngs technology, CSF PCT, and lactate test can assist in diagnosis.

### Sample Collection and Index Detection

Patient sex, age, postoperative temperature, operation time, Intraventricular drainage duration, Intraventricular irrigation, diabetes, pneumonia were obtained. CSF samples were obtained through ventricular drainage. All samples included the last cerebrospinal fluid from which the drainage tube was removed, and the test items included CSF bacteria culture, white blood cell count in CSF, neutrophil ratio in CSF, number of red blood cells in CSF, glucose content in CSF, protein content, and chloride content in CSF. All biochemical tests were performed using standard automated laboratory methods.

### Statistical Analysis

Differences in baseline characteristics between the two groups were determined using spss22.0 for intergroup contrast. Lasso regression was performed for risk factor selection and selected risk factors were used in multivariate analysis. A risk prediction model of secondary CNS infection after ventriculomegaly drainage for intracerebral hemorrhage was established using the RMS R package based on findings from logistic regression analysis. Concordance index (c-index) was used to evaluate the accuracy of the model. Bootstrap method (1000 random samplings) was used to internally validate the model and a calibration curve was generated. The accuracy of the nomogram was explored using Hosmer–Lemeshow test [10]. Area under the curve (AUC) of the ROC curve for the nomogram for prediction of the risk of secondary CNS infection was calculated to determine the discrimination of the model. Clinical decision curve analysis (DCA) was used to explore clinical significance of the model, and a clinical impact curve was generated. Differences were considered significant at a two-sided *P* < 0.05.

### Declarations

All procedures performed in studies involving human participants were in accordance with the ethical standards of the institutional and/or national research committee and with the 1964 Helsinki declaration and its later amendments or comparable ethical standards. This article does not contain any studies with animals performed by any of the authors. For this type of study, formal consent is not required.

### Data Accessibility

The data that support the findings of this study are available on request from the corresponding author. The data are not publicly available due to privacy or ethical restrictions. All patients gave their full consent to participate in this study, and a written consent form was obtained from each patient.

## Results

### Basic Clinical Features

A total of 185 patients were included in the current study, and grouped to CNS infection group (20 patients) and non-CNS infection group (165 patients). CNS infection group comprised 14 males and 6 females, with a mean age of 59.35 ± 9.89 years. The mean operative time of the CNS infection group was 2.18 ± 1.33 h, mean Intraventricular drainage duration was 22.50 ± 14.18 days, mean postoperative temperature was 38.77 ± 0.4964 °C, WBC count was 18009.30 ± 24567.19 × 10^6^/L, neutrophil ratio was 87.05 ± 8.42%, RBC count was 216045.00 ± 314093.96/L, CSF glucose content was 1.26 ± 1.12 mmol/L, CSF protein content was 4.59 ± 3.58 g/L, and CSF chloride content was 119.26 ± 9.24 mmol/L. The non-CNS infection group comprised 97 males and 68 females with an age of 60.95 ± 12.37 years. In addition, the mean operative time of the non-CNS group was 1.78 ± 1.27 h, mean Intraventricular drainage duration was 13.16 ± 9.71 days, mean postoperative temperature was 38.13 ± 0.76 °C, WBC count was 1551.23 ± 3912.15 × 10^6^/L, neutrophils ratio was 69.49 ± 24.11%, RBC count was 145736.48 ± 404144.48/L, CSF glucose content was 3.53 ± 1.61 mmol/L, CSF protein content was 4.35 ± 5.36 g/L, and CSF chloride content was 121.46 ± 6.82 mmol/l (Table 1).

**Table 1:** Basic clinical characteristics of the two groups

Demographic characteristics	CNSIs (NO, *n* = 165)		CNSIs (YES, *n* = 20)		Total (*n* = 185)	
Sex, *n* (%)						
Male	97	58.79%	14	70.00%	111	60.00%
Female	68	41.21%	6	30.00%	74	40.00%
Age	60.95 ± 12.37		59.35 ± 9.89		60.77 ± 12.11	
Operation time, *n* (%)	1.78 ± 1.27		2.18 ± 1.33		1.82 ± 1.27	
Intraventricular drainage duration	13.16 ± 9.71		22.50 ± 14.18		14.17 ± 10.64	
Intraventricular irrigation, *n* (%)						
Yes	89	53.94%	15	75.00%	104	56.22%
No	76	46.06%	5	25.00%	81	43.78%
Postoperative temperature	38.13 ± 0.76		38.77 ± 0.50		38.20 ± 0.76	
White blood cell count in CSF, *n* (%)	1551.23 ± 3912.15		18,009.30 ± 24,567.19		3330.48 ± 10,110.56	
Red blood cell count in CSF, *n* (%)	145,736.48 ± 404,144.48		216,045.00 ± 314,093.96		153,337.40 ± 395,279.18	
Neutrophil ratio in CSF, *n* (%)	69.49 ± 24.11		87.05 ± 8.42		71.39 ± 23.57	
CSF glucose content, *n* (%)	3.53 ± 1.61		1.26 ± 1.12		3.29 ± 1.72	
CSF protein content, *n* (%)	4.35 ± 5.36		4.59 ± 3.58		4.37 ± 5.19	
CSF chloride content, *n* (%)	121.46 ± 6.82		119.26 ± 9.24		121.22 ± 7.13	

### Lasso Screening for Associated Risk Factors

**Fig. 1:**
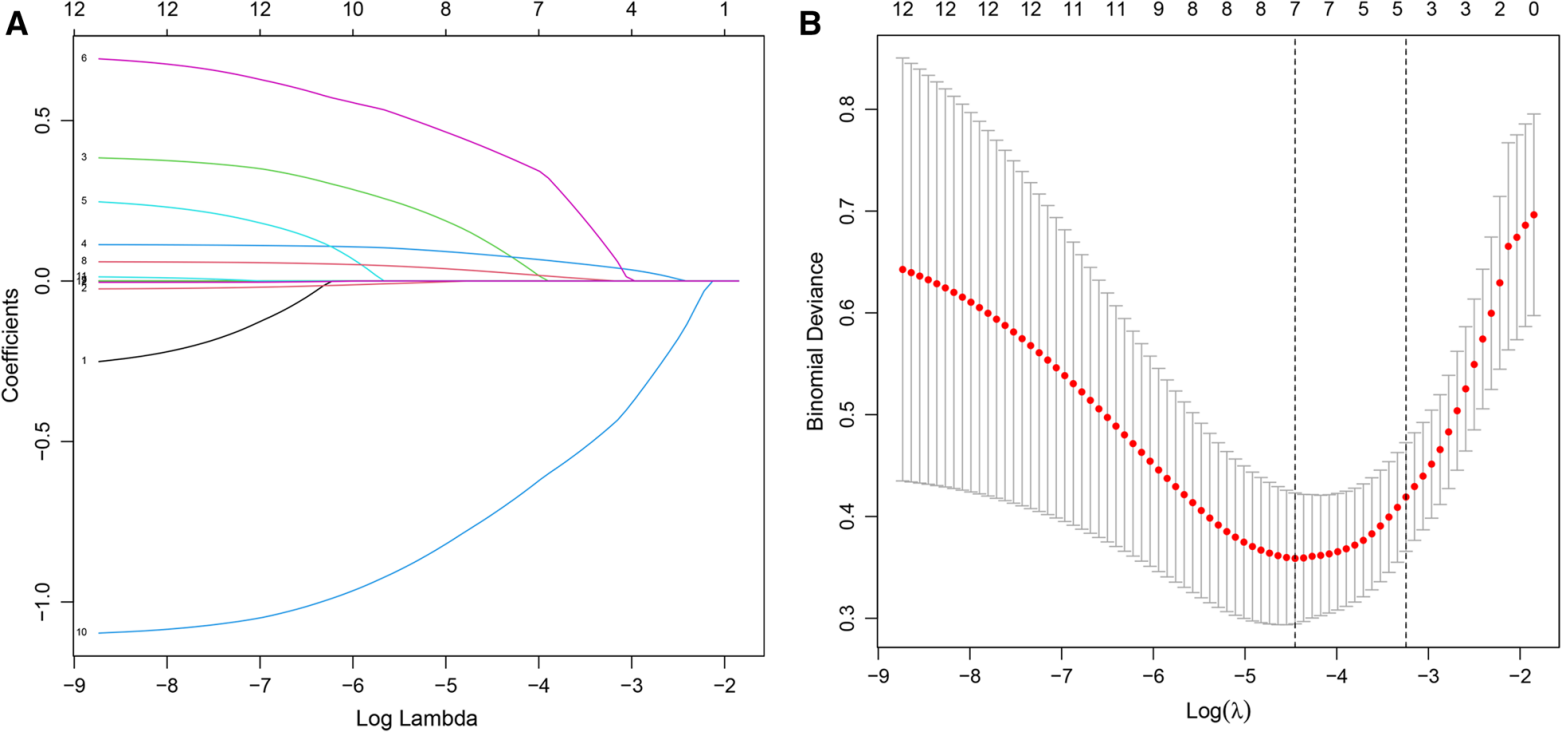
Lasso regression analysis. **A** Lasso regression curves, where each curve corresponds to one variable. None of the regression parameters was zero. **B** Fivefold cross validation plot with log on the horizontal axis (*λ*) values, the uppermost number indicates the number of included variables. The dotted left line corresponds to the lowest point of the red curve. The dotted line on the right corresponds to the simplest model

Lasso regression was used to screen risk factors that are potential predictors of CNS infection. Nine variables, including operative time, Intraventricular drainage duration, postoperative temperature, CSF WBC, CSF neutrophils ratio, CSF RBC, CSF glucose content, CSF protein content, and CSF chloride content, were used in constructing the lasso regression curve using R glmnet package (Fig. 1a). The fivefold cross validation plot is shown in Fig. 1b. The dotted left line indicates the lowest point of the red curve, which corresponds to *λ* values for optimal *λ* value, which has the lowest model error at this time. The simplest model was obtained within one standard deviation of the dotted left line, corresponding to the dotted right line. A total of 7 variables including operative time, Intraventricular drainage duration, postoperative temperature, CSF WBC count, CSF neutrophils ratio, CSF RBC, and CSF glucose content were included in the analysis.

### Logistic Regression Analysis and Nomogram Construction

**Fig. 2:**
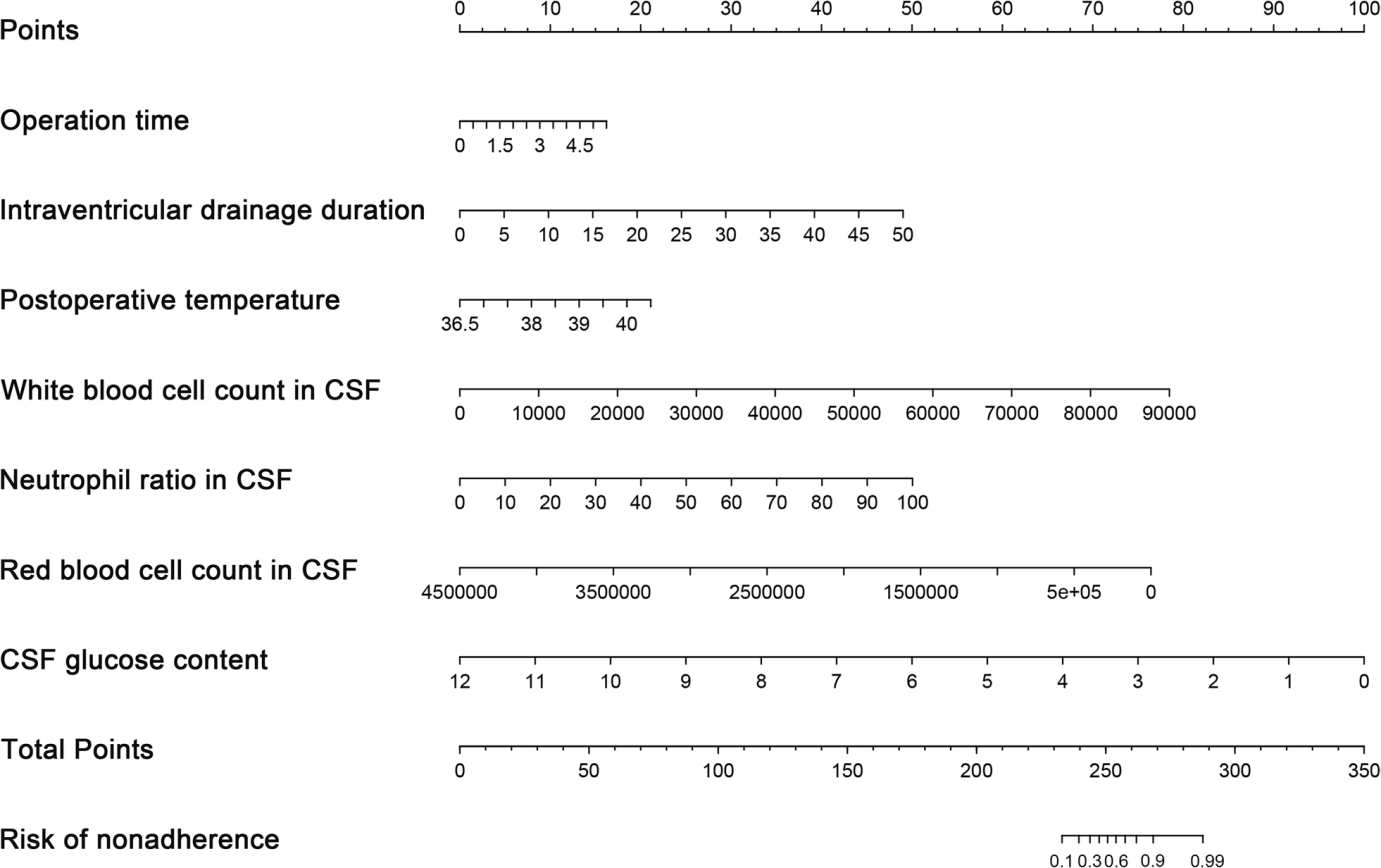
The uppermost scale is the score corresponding to each variable, the scores of each variable are summed, and the resulting total score corresponds to the lowest axis of probability values to obtain the probability of secondary CNS infections

Seven variables screened by lasso regression were included in multivariate logistic regression analysis. The findings showed that intraventricular drainage duration (*P* = 0.0032) and CSF glucose content (*P* = 0.01) were independent risk factors for predicting secondary CNS infection in patients who underwent ventriculostomy drainage for ICH. Notably, the risk of CNS infection increased with increase in the Intraventricular drainage duration and with decrease in CSF glucose level. Lasso regression findings were combined with clinical characteristics to construct a nomogram model based on logistic regression model. The nomogram comprised 7 variables including operative time, intraventricular drainage duration, postoperative temperature, CSF WBC count, CSF neutrophils ratio, CSF RBC count, and CSF glucose content (Fig. 2). The odds ratio values for each variable are shown in Table 2. The findings showed that the longer operative time, longer intraventricular drainage duration, higher the temperature, higher CSF white blood cell count, higher CSF neutrophil ratio, lower CSF red blood cell count, and lower CSF glucose content were all correlated with higher risk of secondary CNS infection. Each variable that corresponded to the score of the upper scale was summed, and the resulting total score indicated the most inferior axis of probability values to obtain the probability of having a secondary CNS infection, which is shown on the Fig. 2 as risk of nonadherence.

**Table 2:** Odds ratio values for 6 variables

Intercept and variable	Prediction model		
	B	Odds ratio (95% CI)*	*P*
Intercept	− 33.26	3.60E – 15 (3.03E − 38–8.760027E10)	0.19
Operation time	0.37	1.44 (0.74–2.93)	0.28
Intraventricular drainage duration	0.12	1.13 (1.05–1.24)	0.0032
Postoperative temperature	0.66	1.93 (0.58–7.37)	0.30
White blood cell count in CSF	1.09E − 4	1.00 (1.00–1.00)	0.07
Neutrophil ratio in CSF	0.06	1.06 (1.00–1.18)	0.12
Red blood cell count in CSF	2.11E − 6	1.00 (1.00–1.00)	0.43
CSF glucose content	− 1.04	0.35 (0.14–0.70)	0.01

*Compared via the odds ratio

**Fig. 3:**
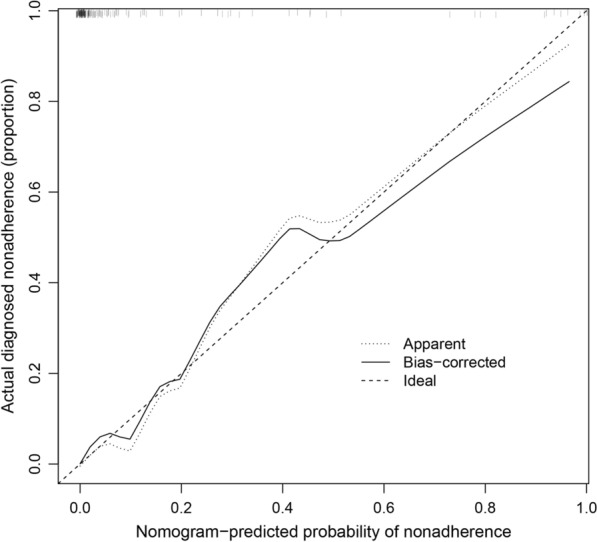
Calibration curve for prediction of CNS infection after ICH

For example, for a patient with intracranial hemorrhage, the operation took 2 h, the postoperative temperature was 36.5 degrees, the ventricle was drained for 5 days, the white blood cells in cerebrospinal fluid were 4 × 10^4^, the proportion of neutrophils in cerebrospinal fluid was 20%, the red blood cells in cerebrospinal fluid were 2.5 × 10^6^, and the glucose in cerebrospinal fluid was 7 mmol/L, so the total score of the patient on the model was about 156. According to further calculation, the probability of CNS complications in this patient was 32%. Therefore, this patient does not need to be treated with CNSI. Moreover, the model showed a c-index of 0.961 indicating a good predictive ability for CNS infection. Bootstrap method (1000 random samplings) was used to internally validate the model and for construction of the calibration curve (Fig. 3). The calibration curve was approximately a straight line with a slope close to 1, indicating high accuracy of the model in predicting postoperative CNS infection after intracerebral hemorrhage.

### ROC Curve and DCA

**Fig. 4:**
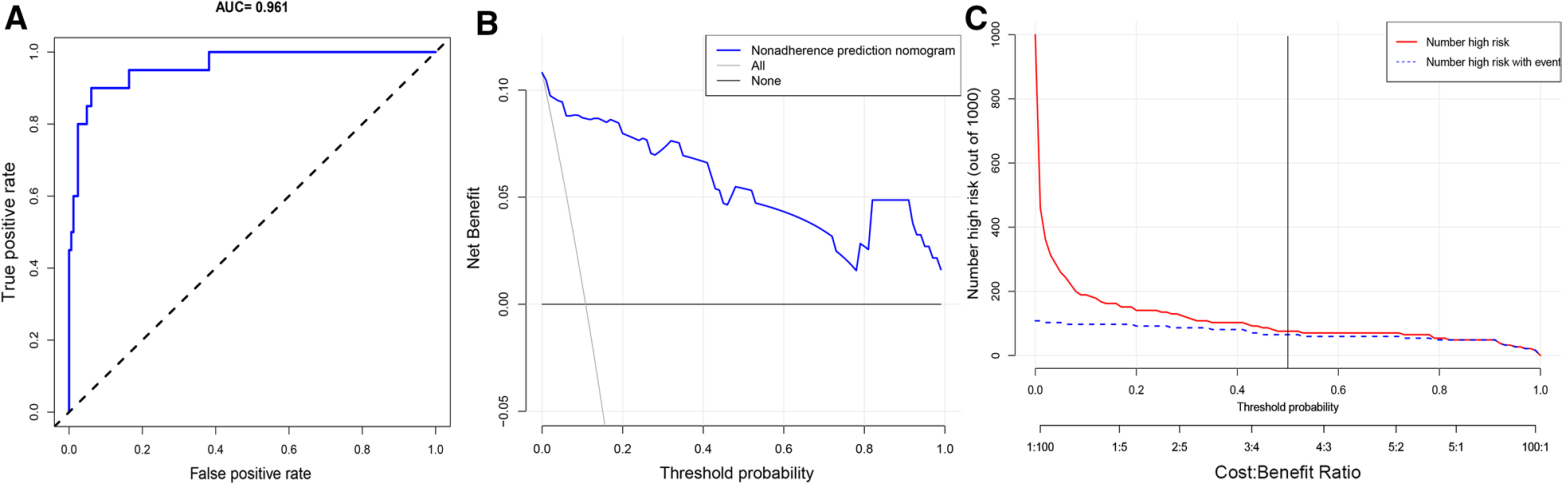
ROC curve and DCA. **A** ROC curve to evaluate discrimination of the nomogram model. **B** DCA to evaluate if the nomogram improves clinical decision-making. **C** Clinical impact curves of the nomogram for distinguishing CNS infections

To explore the ability of the model to accurately predict whether CNS infection was secondary, ROC curves were generated. Analysis of the ROC curve showed that the model had an AUC of 0.961 for predicting CNS infection after ICH (Fig. 4a). The high AUC indicates that this nomogram model had a good discrimination. DCA was used to evaluate whether the nomogram can improve clinical decision-making. Analysis of the decision curve (Fig. 4b) showed that the nomogram had better application value when the risk predicted by the model was between 1 and 100%. The abscissa of figure B is the threshold probability: in the risk assessment tool, the probability of patient I diagnosed with membranous nephropathy is recorded as PI; When PI reaches a certain threshold (recorded as Pt), it is defined as positive and treatment measures will be taken. At this time, there will be benefits (advantages) of patient treatment, injuries not treated by patients and losses (disadvantages) not treated by patients. The ordinate is the net benefit (NB) after subtracting the advantages from the disadvantages. One oblique line in Figure B represents the clinical diagnostic model of CSF, respectively. In addition, there are two lines, which represent two extreme cases. The horizontal one represents that all samples are negative (PI < PT), no one is treated, and the net benefit is 0. The oblique one indicates that all samples are positive, all have received treatment, and the net benefit is a backslash with a negative slope. Next, the clinical impact curve was generated to analyze the number of high-risk patients and the number of high-risk patients with CNS infections at different threshold probabilities (Fig. 4b). We comprehensively consider DCA and clinical impact curve to make a balance between higher net benefit and lower false-positive rates. The Fig. 4b and c showed that when the risk of implantation failure threshold is set at 0.50, it provides the exceeding low false-positive rate and significant clinical benefit to the entire included population.

## Discussion

Volume of intracerebral blood is an important factor affecting prognosis of patients with intracerebral hemorrhage. Therefore, the most preferential treatment for patients with intracerebral hemorrhage should be prompt removal of intracerebral hemorrhage [11]. In addition, extracerebral drainage relieves the space occupying effect of hematoma, reduces intracranial pressure, and reduces occurrence of obstructive hydrocephalus [12]. However, the CNS infection rate after neurosurgery is 4.6–25% [13], accounting for 0.8–7% of CNS infections [14]. Incidence of extraventricular drainage-related infection ranges from 8 to 22% [15]. Extraventricular drainage is a major risk factor for neurologic infections [16].

Secondary CNS infections after ventriculostomy tube drainage, if not treated promptly at an early stage, often lead to patients having CNS sequelae and may cause disability and death [17, 18]. Therefore, early diagnosis is extremely important for early prediction of patients with secondary CNS infections. However, cranial CT and MRI are not specific for meningitis. Moreover, CSF bacterial culture has several disadvantages such as low sensitivity and it is time-consuming to culture samples. Therefore, there is an urgent need to find tools that can improve early diagnosis of CNS infections.

CNS infections include meningitis and (or) encephalitis, mainly caused by abnormal cerebrospinal fluid such as an elevated white blood cell count [19]. CSF in the acute phase shows cloudy, yellow, or purulent changes in most patients with CNS infection. Postoperative temperature and CSF white blood cell count have been used for diagnosis of cerebrospinal fluid infection [20]. However, CSF white blood cell count (WBC) > 10 × 10^6^/L, neutrophil ratio > 70% is one of the main diagnostic criteria for CNS infection. And red blood cells in CSF caused by traumatic tap or subarachnoid hemorrhage artificially increase white blood cell count and protein levels, thus confusing the diagnosis [21]. Furthermore, fever (body temperature > 38 °C) is a diagnostic basis of CNS infection [16]. The findings of the current study showed that high temperature of the patient, high CSF white blood cell count, and high CSF neutrophil ratio, were correlated with increased risk of secondary CNS infection, which is consistent with findings from previous studies and our clinical experience. In addition, patients are at significantly increased risk of developing CNS infections as the number of days of tube placement increases [22]. A previous prospective study reported that an Intraventricular drainage duration ≥ 8 days is correlated with higher risk of CSF infection [23]. Surgery times > 4 h significantly increase incidence of neurological infections in patients [24]. The findings of the current study showed that intraventricular drainage duration (*P* = 0.013) was an independent risk factor for predicting secondary CNS infection in patients with ICH who had undergone ventriculostomy drainage. Notably, the risk of secondary CNS infection increased with increase in intraventricular drainage duration. This indicates that surgeons should have good control over operative time when performing ventriculoperitoneal drainage. Normal concentration of CSF glucose is 2.5–4.5 mmol/L, which is 2/3 of the serum glucose level [25]. The findings of the current study showed that CSF glucose content (*P* = 0.0075) was an independent risk factor for predicting secondary CNS infection. This is consistent with the findings by Namani et al. that reduced CSF sugar levels indicate a poor prognosis in patients with septic meningitis [25]. The bacteria responsible for the patient’s secondary CNS infection may have increased anaerobic respiration and consumed the sugars in the CSF, thus reducing CSF sugar level.

## Conclusion

In summary, the findings of the study showed that operative time, intraventricular drainage duration, postoperative temperature, CSF white blood cell count, CSF neutrophils ratio, CSF red blood cell count, and CSF sugar content were risk factors for secondary CNS infection. A nomogram was established based on the above-mentioned risk factors to predict the risk of secondary CNS infection after ventriculomegaly catheterization for cerebral hemorrhage with good discrimination and accuracy. Notably, the nomogram is simple and easy to perform, and the indicators used are all clinically available items. However, this study is a single center study with a relatively small sample size. Therefore, a large sample multicenter study to provide more information for risk prediction of CNS infections should be conducted. However, this model provides a basis for clinical management of secondary CNS infections after ventriculomegaly drainage. In addition, it helps guide clinicians in screening high-risk patients.
